# Cuicatec ethnozoology: traditional knowledge, use, and management of fauna by people of San Lorenzo Pápalo, Oaxaca, Mexico

**DOI:** 10.1186/s13002-019-0340-1

**Published:** 2019-11-27

**Authors:** Leonor Solís, Alejandro Casas

**Affiliations:** Instituto de Investigaciones en Ecosistemas y Sustentabilidad (IIES), Universidad Nacional Autónoma de México (UNAM), Campus Morelia, Antigua Carretera a Pátzcuaro No. 8701, C.P, 58190 Morelia, Michoacán Mexico

**Keywords:** Animal management, Biocultural heritage, Biodiversity conservation, Cuicatec, Ethnozoology, Tehuacán Valley

## Abstract

**Background:**

The Tehuacán-Cuicatlán Valley is a region of outstanding biocultural diversity, harboring eight indigenous ethnic groups and a remarkable biodiversity in a territory 10,000 km^2^ extent. Ethnobotanical studies of the region are among the most complete in Mexico; contrarily, ethnozoological studies are still limited. But information on both flora and fauna use and management is relevant for understanding local cultural and ecological issues, and for planning integral strategies of biodiversity conservation. Our study focused on analyzing knowledge and use of animals and their relationship with faunistic management by the Cuicatec, an ancient human culture whose distribution is restricted to the region. We hypothesized that wild animals still have significant contributions to diet, medicine, and spiritual life of the Cuicatec people. In addition, we expected to find a gradient of interactions, from simple gathering and hunting to communitarian regulations of use, specialized management techniques and care, nurturing, and domestication of animals. Such gradient of management interactions would be influenced proportionally with cultural and economic values, viability maintenance, and scarcity of animals.

**Methods:**

Our study was carried out in San Lorenzo Pápalo, Oaxaca. We conducted surveys and semi-structured and open interviews to people to document the Cuicatec nomenclature, classification, use, and management of fauna, as well as their perceptions about abundance, risks of disappearance, and availability of wild animals. We used images of animal species reported for the area as communication stimuli for confirming their local presence. Also, we recorded skins and skulls used as trophies and ornamental objects, pawprints, and excretes. Through free listing, we identified the most meaningful species of different animal groups. Whenever possible, we evaluated amounts of animals obtained from the wild, and for some species, we compared this information with data on their distribution and abundance evaluated through ecological sampling, to explore indicators on their sustainable use.

**Results:**

The Cuicatec name all animals through the term *i-ti* and classify them in several groups of vertebrates, arthropods, and mollusks, some of them coinciding with the formal taxonomy and some others based on their social-cultural role. The most meaningful animals are 23 species of edible organisms, outstandingly the *chicatana* ants (*Atta mexicana*) and the *cuetla* Lepidoptera larvae (*Arsenura armida*), the lizard *Sceloporus grammicus*, and among the mammals some squirrels (*Sciurus* spp.), badgers (*Nasua narica*), and deer (*Odocoileus virginianus*). Some species were reported to be used for medicinal purposes, among them opossum (*Didelphis* spp.) and macaws (*Ara militaris*), used to ease childbirths, but this use almost disappeared. Local perception of availability of animal resources is associated to forest conservation. Regulations for protecting forests and the most used animal species were recorded; the rules are mainly associated to hunting and gathering seasons, respecting females of vertebrate species, and permits for gathering and hunting given by local authorities. Nurturing of animals was recorded in bird and mammal species, but in no case, their breeding was achieved.

**Conclusions:**

Animals are important elements of the Cuicatec culture and subsistence, complementing their diet based on agricultural products. Animals used as medicine were still reported but substituted by modern medicine. There is a consensus about the need to conserve forests to ensure the maintenance of animals, which are valued as part of nature, the beauty of their territory, and culture. Communitarian regulations are the main ways for conserving fauna, but local techniques of animal management may help in designing conservation strategies.

## Background

The Tehuacán Valley has been recognized because of its outstanding biocultural diversity. Its territory, 10,000 km^2^ extent, is inhabited by eight indigenous ethnic groups: the Mixtec, Nahua, Chocho, Popoloca, Mazatec, Ixcatec, Chinantec, and the Cuicatec [1]. In addition, this region harbors a high biodiversity. Researchers have reported more than 3000 species of vascular plants [2], and although information on fauna is still limited [3], the partial information available for some groups reveals that diversity of animals is also remarkable. For instance, Brailovsky et al. [4, 5] reported 24 species of the hemipteran Coreidae, Ayala et al. [6] recorded 36 species of bees, and Ríos-Casanova et al. [7] registered 28 species of ants. For vertebrates, Canseco and Gutiérrez-Mayén [8] reported 32 and 117 species of amphibians and reptiles, respectively, whereas Arizmendi and Espinoza de los Montero [9] and Arizmendi and Valiente-Banuet [10] recorded 150 species of birds, and Téllez-Valdés et al. [11] 98 species of mammals. The fauna inventory of the region has substantially increased during the last 15 years, but it is far to be complete. Ethnozoological information is even scarcer, and it is a research priority [2, 3, 12], since information from this field will be necessary for an integral understanding of regional cultural and ecological issues. It would be crucial for planning conservation strategies and for controlling factors that affect animal populations and determine risk to their permanence in the region [1, 3, 9, 12, 13]. Among other issues, it is relevant for the evaluation and regulation of hunting and extraction of some species [1, 3, 12, 13], protecting bats from disturbance and irresponsible policies for controlling cattle rabies [1, 3, 11–13], as well as protecting numerous species of pollinators against the uncontrolled use of pesticides [1, 3, 13].

The long history of interactions between humans and animals in the region [1–3] is currently reflected in a deep indigenous knowledge of diversity, properties, benefits, damages, interactions, symbolism, myths and customs, practices for using them, and for promoting or controlling their abundance [14]. All these aspects are main expressions of the traditional ecological knowledge (TEK) of peoples of the area. Documenting and understanding human culture in relation to the TEK, management and worldviews in relation to animals are main goals of ethnozoology.

Archeological studies in the Tehuacán Valley allowed reconstructing the most complete stratigraphic chronology of the prehistory of Mesoamerica [15, 16]. These studies revealed that during the early occupation of the region by humans, hunting was the main activity and, even after the extinction of the megafauna, people continued being mainly hunters. They then combined their subsistence with progressively more intense gathering of plants and, eventually, domestication of some plant species [2]. Among the earliest remains of animals associated to human subsistence are those identified by Flannery [17] (Appendix). TEK about fauna, management techniques, and the role of animals in worldviews are all aspects for establishing important bases for sustainable use and conservation of fauna in the region [1–3, 12].

Animals are primary sources of food, but they also provide medicines and other goods [18, 19]. Documenting use of animals in the Tehuacán Valley is one of our main concerns in this and other on-going studies, but in addition, the forms and intensities of management interactions local people establish with fauna and the reason of the intensity of such interactions that may include from simple gathering to domestication [14].

Animals are fundamental components of natural ecosystems and responsible of crucial functions like herbivory, predation, pollination, seed dispersal, degradation of organic matter, soil removal and aeration, among others. Animals have therefore provided important resources and environmental benefits to humans. Numerous species of arthropods and vertebrates are used as food by peoples throughout the world [20]; others are medicines [21], ornamental, and raw matter for handcrafts [22–24]. Thousands are important pollinators, seed dispersers, or pest-controlling agents of crops [25–27]. Many species determine risks to human health since they are parasites, and vectors of dangerous illnesses [28], while others cause conflicts because they consume crops or predate domestic animals [29–31].

It is currently recognized that animals are and will be important in programs of food security and sovereignty [32], which are priority topics of research for the contemporary global science [33], and ethnobiology has a high promising value for inventorying food and techniques that can be potentially included in this task. But studying management of animals has theoretical importance. Our research team has constructed theory about factors influencing different states and intensities of interactions between people and plants. In those studies, we identified simple, planned, and selective gathering, let standing of desirable species and phenotypes when clearing areas for agriculture, enhancing, transplanting, and cultivation of plants through vegetative propagules or seeds, and domestication involving intensive human selection [1, 2]. Those studies aspire contributing to understand factors explaining routes to domestication and origins of agriculture. But in Mesoamerica, animals, fungi, and microbiota have been also managed by humans. Understanding forms of interactions between humans and different groups of organisms and the factors influencing such interactions are therefore important topics for understanding agriculture [1, 2, 34, 35].

Numerous ethnobiological studies have investigated and constructed theories about how peoples of the world practice hunting, gathering, nurturing, husbandry, and domestication of plant and animal resources [14, 22–24, 34, 35]. Wild animal resources directly used, but also interchanged and traded [24, 36]. Frequently, rural people have been blamed to be responsible of decreasing forest cover and fauna populations; however, it is now clear that the most powerful processes destroying natural ecosystems at global scale are the industrial processes of production in both rural and urban areas and that these have been particularly accelerated after the 1940s [37]. Currently, it has been recognized that several species are endangered because of hunting [38] and over-exploitation of some species due to their medicinal use. However, these are particularly the cases of perverse nets of trading mafias rather than practices for rural subsistence [36, 39, 40]. Deforestation, the exponential increasing of grasslands, the intensification of agriculture through chemical inputs, the expansion of urban areas, and the dangerous excretions of industries are all the most significant causes of the global change and degradation of the natural ecosystems over the world [23, 41, 42]. Conservation policies are unable of controlling the huge destruction of ecosystems by industry, but paradoxically, some conservationists deny the possibilities of obtaining benefits of traditional cultural importance associated to using biodiversity and ecosystems for satisfying basic needs of indigenous peoples [20]. Therefore, understanding the way local peoples use and maintain ecosystems is nowadays a crucial issue for conservation. Documenting traditional knowledge, use, and management of natural resources provides not only information about actual and potential resources for solutions of global problems such as food security, but in addition, valuable techniques of sustainable management for ensuring the maintenance of biodiversity and ecosystems [1, 13].

Traditional ecological knowledge of Mesoamerican peoples is among the most remarkable, because of their long history and the high diversity of contexts, from Mexico to Costa Rica [43], with peoples that have historically based their subsistence on agriculture, use of non-timber forest products, and raising of animals. The latter activity has increased its importance after the Spanish Conquest. The traditional Mesoamerican systems of subsistence have elements in common, but particularities marked by their ecological, cultural, and historical contexts [12]. One common characteristic among the Mesoamerican cultures is the extended practice of entomophagy, apparently more marked than in other regions of the Americas, where animal proteins in food were obtained from hunting (like in North America and the Amazonia), or from a more developed pastoralism and raising of animals like in the Andean region [14, 44]. Wild animals and insects and other arthropods have been part of diet of Mesoamerican peoples, who consider them healthy, tasty, and nutritious, since they are sources of good quality protein [20].

Our study focused on a Mesoamerican culture, the Cuicatec, which inhabit the southeast of the Tehuacán-Cuicatlán Valley [45]. The Cuicatec is an Otomanguean language closely related with the Mixtec [46]. It is one of the least known indigenous peoples of Mexico, and one of the least studied from ethnobiological approaches. This is in part due to their isolation, since the Cuicatec villages are 2 to 5 h through rustic roads from the main town Cuicatlán, and these rustic roads were inexistent 35 years ago. In addition, the Cuicatec are distributed in a relatively small area (17 villages) with few people (nearly 13,000 people in total) compared with other indigenous groups of Mexico [47, 48].

We documented knowledge, use, and forms of interaction and management of animals by the Cuicatec of the village of San Lorenzo Pápalo, Oaxaca. Our previous studies in the region [1, 45] documented that domestic animals are mainly a way of saving money for households which are sold in cases of emergency or consumed during feasts. Therefore, wild fauna should play an important role in the daily life diet and intake of animal protein. We expected to find a gradient of interactions from simple forms of gathering and hunting to husbandry and domestication. We hypothesized that wild animals still have significant contributions in diet, medicine, and spiritual life of the Cuicatec. In addition, we expected to find a gradient of management intensity from simple gathering and hunting with no procurement nor care of animals, to communitarian regulations for use strategies, management techniques, special care and husbandry, and domestication. We supposed that intensity of interactions would be proportional to their cultural and economic value, the ease to maintain them in human-controlled areas, their scarcity, and uncertainty in their availability.

## Methods

### Study area

San Lorenzo Pápalo belongs to the municipality of Cuicatlán, Oaxaca, in the Tehuacán-Cuicatlán Valley. The village is settled into elevations averaging 1900 m, but the territory of the community covers a range from 800 to 3000 m, with tropical dry forest in the lowlands, pine-oak forests, and patches of cloud forests in the highlands [12]. The village is approximately 4 h far from the main town Cuicatlán, through a rustic road. There are an ancient church, a small health center attended by one physician, and primary and secondary school, and it is inhabited by nearly 120 households with nearly 600 persons, all of them Cuicatec. All people speak Cuicatec in their daily life but most of them are bilingual, speaking Cuicatec and Spanish. Climate in the lowlands is warm and dry, whereas in the highlands is temperate. Annual precipitation in the area where the village is settled is on average 1090 mm and temperature 16 °C [12]. The community used to practice agreements on timber extraction with private companies until 15 years ago, when they decided to stop those agreements that severely damaged their communitarian territory with very poor gains for local people [12]. Since then, the forests have partly recovered. People live mainly from maize agriculture, complementing their economy with production of fruit in homegardens (mainly *granadilla*, *Passiflora edulis*), goat and cattle raising, and extraction of non-timber forest products [12]. Migration to cities of Mexico and to the USA is a complementary source of income. It is a well-organized community based on the traditional customs regime [12].

### Ethnozoological studies

For documenting the Cuicatec nomenclature and classification of vertebrates, arthropods, and other invertebrates, we conducted semi-structured interviews to people. We used images of animals of different groups reported for the area as stimuli, and some others were directly photographed. We in addition identified skins and skulls kept by people as ornaments and trophies, as well as pawprints and excretes of some species. Other animals were referred to by people when we asked about similar animals to those of a specific group. Animals referred to in the interviews were identified based on regional inventories and guides, EncicloVida, an important digital platform, and the National System of Biodiversity Information constructed by the National Commission for the Knowledge and Use of Biodiversity (CONABIO), Mexico. We conducted open interviews to document animals and names belonging to different groups. Then, we carried out semi-structured interviews for documenting more particular groups and recording detailed information about names, classification, forms of use, and management. We continually compared the information obtained in the field with information recorded in the Cuicatec-Spanish Dictionary by Anderson and Roque [49] based on information documented in the neighboring village of Santa María Pápalo. In addition, we carried out surveys with 30 households (nearly 25% of all households in the village) to collect deeper information on local forms of use and management of fauna. The surveys were designed for particular species, including questions on amounts of products obtained throughout the year, gathering or hunting seasons, sites and techniques of hunting and collection, perception about disappearing risk of the species used, and factors influencing that risk. Based on information about amounts consumed per household, we estimated the amounts of the main animal species consumed by the whole village per year. These estimations were compared with the general patterns of local diet previously documented by a survey to the households included in this study [12].

In addition, we conducted semi-structured interviews [50, 51] to local authorities and households of the village to document the perception of local people about threatens of fauna. Also, we documented the communitarian regulations and actions collectively constructed to protect fauna and the mechanisms for ensuring the fulfillment of agreements.

### Data analyses

For the most consumed species, we estimated the biomass annually consumed by households throughout the year. For those estimations, we identified different measurement units mentioned by people in the interviews. Whenever possible, we directly weighted samples of those units to estimate the average weight of animals consumed, multiplied by the times people consume a species during the season they are available.

For the *cuetla* or *jonocuil* (larvae of Saturnidae butterflies), we estimated the balance between amounts consumed and amounts of larvae available. This was possible since the larvae infest specifically the *jonote* tree (*Heliocarpus velutina*), and we counted the number of trees and all larvae occurring on the trees in a 500-m^2^ sampled area. We could estimate the total amount of larvae available in patches of tropical dry forest where the *H. velutina* occurs in the territory of San Lorenzo Pápalo. We used this case study as a model for discussing how to evaluate the risk of hunting, trapping, or gathering wild animals or their products.

## Results

### Cuicatec nomenclature and classification of animals

The Cuicatec term for animal is *i ti*, which designates a broad spectrum of organisms of the animal kingdom, and the prefix is used for naming different groups of animals. For instance, *i ti chi snen gue che* is used for designating all birds, *i ti chin yita vi ya* for all domestic animals, *i ti gue ie yuta* for naming some mammals, *i ti lín* for all insects, and *i ti tan* for all “fierce animals” (mainly carnivore species)*.*

Among the invertebrates, we identified nine terms grouping several species of insects each. One of them is the term *c’udi*, which would be generally similar to bug, but we recorded within it some ants, including the *chicatanas* ants (*Atta cephalota*) that are highly appreciated as food and that are called *tu c’udi* in Cuicatec. Another group of insects is called *i’cu*, which includes lice, termites, and other species of ants different to *chicatanas*. And other groups use the prefix *i ti*, including insects like dragonflies, arachnids like scorpions, and snails (Table 1). Some differences between the Cuicatec-Spanish dictionary and nomenclature recorded in the community studied are indicated in Table 1. For instance, beetles and isopods are named with the prefix *nd* as well as wasps and bees that in San Lorenzo Pápalo are named with the prefixes *sti’ gua* and *tu mín*, respectively. The prefix *nd* is used for naming worms, caterpillars*,* and some beetles. However, this term establishes differences in the generic names; for instance, the group *nd’a* or *nd’oo* refers to worms and caterpillars, *nd’i* to beetles, and *nd’u* to isopods (*nd’oo ya’a dacua* is, for instance, the name of the edible larvae or worm of the *jonote* tree). Another group of invertebrates is named with the prefix *san*, mainly used for fireflies and dragonflies, as well as crabs. Some examples are shown in Table 2. The term *sti* is used as prefix for naming wasps and cockroaches, while the term *tu* groups grasshoppers, some non-edible ants and bumble bees. The prefix *y’a* groups forms of winged insects such as mosquitoes, flies*,* and bees, while in Santa María Pápalo bees are grouped by the term *y’en*. In Santa María, spiders are grouped with the term *y’a nini*, while in San Lorenzo these organisms are classified with the term *sa ibi*. Butterflies are generally grouped with the term *y’a va* in Santa María and *y’evi* in San Lorenzo. Several groups are diffuse in their classification. These are the cases of creeks (*y’undi*), fleas (*‘i yu*), bedbugs (*‘i yun*), and large mosquitoes (*quen*).

**Table 1:** Examples of Cuicatec nomenclature and classification of invertebrates, based on fieldwork in San Lorenzo Pápalo and reports from [49] in Santa María Pápalo, Oaxaca

Common name	Scientific name	Generic term	Specific term
*ICU* Group			
*Piojo negro*	*Pediculus humanus* var. *capitis*	*icu tin*	
*Cochinilla*	Isopoda *Armadillium* sp.	*icu tin*	*tna’an*
*Piojo blanco*	*Pediculus humanus* var. *corporis*	*icu tin*	*tinu*
*Piojo del marrano*	*Haematopinus suis*	*icu ye’en*	*cuchi*
*Piojo de la gallina*	*Dermanyssus gallinae*	*icu ye’en*	*tu’u*
*Hormiga arriera*	*Atta mexicana*	*icu*	
*Hormiga arriera*	*Atta cephalota*	*icu*	
*Hormiga de fuego*	Hymenoptera-Formicidae	*icu nda*	*a*
*Hormiga alada*	*A. mexicana*	*icu nda*	*a gueche*
*Hormiga de hueso*	Hymenoptera-Formicidae	*icu nda*	*ini*
*Hormiga de palo seco*	Hymenoptera-Formicidae	*icu nda*	*a langu*
*Hormiga de humedad*	Hymenoptera-Formicidae	*icu nda*	*a nuni*
*Hormiga barredora*	Hymenoptera-Formicidae	*icu nda*	*a tan*
*ITI* Group			
*Chahuistle*	*Ventuna inaequalis*	*iti dan*	
Scorpion	*Centruroides* sp*.*	*iti da’a*	
*Tijerilla*	*Forticula auricularia*	*iti dìnda*	
Capuline spider	*Latrodectus mactans*	*iti nd’iya*	
Firefly	*Lampiris noctiluca*	*iti a’an*	
*ND* Group			
Worm, caterpillar	Lepidoptera several species	*nd’a*	*gun cuan*
*Chicatana*	*Atta mexicana* (winged form)	*nd’a a*	*a san ‘go*
Beetles	Coleoptera, several species	*nd’i vi*	
*SAN* Group			
Crab	Gecarcinidae, several species	*san*	*goo*
Dragon fly	*Anax junios*	*san*	*ta jun*
Firefly	*Lampiris noctiluca*	*san*	*dili’i*
*STI* Group			
Red wasp	Hymenoptera Vespidae	*sti*	*gu’a*
Wasps	Hymenoptera Vespidae, several species	*sti*	*nyun*
Cockroach	*Periplaneta americana*	*sti*	*vi*
*TU* Group			
Grasshopper	*Sphenarium purpurascens*	*tu*	*cua*
Bumblebees	*Bombus* spp.	*tu*	*joo*
*Y’A* Group			
Flies	*Musca domestica*	*y’a*	*cu*
Mosquitoes	Culicidae-Several species	*y’a*	*yoo*
Spiders	Araneae-Several species	*y’a*	*ibi*
Bees	*Apis melifera*	*y’a*	*nda’a*
Melliponini bees	Apidae-Meliponini-Several species	*y’a*	*mutu*
Bees	Apidae-Several species	*y’a*	*cúa*
Butterflies	Lepidoptera-Several species	*y’a*	*vi*

**Table 2:** Examples of Cuicatec nomenclature and classification of reptiles, based on fieldwork in San Lorenzo Pápalo and reports from [49] in Santa María Pápalo, Oaxaca

Common name	Scientific name	Generic term	Specific term
Lizards			
White lizard	*Sceloporus grammicus*	*y’ati*	*cua*
Red tong lizard	*Sceloporus* sp.	*y’at*	*dhabi*
*Lagartija del cerro*	*Sceloporus* sp.	*y’at*	*cueé*
Snakes			
Rattlesnake	*Crotalus* spp.	*cu*	*ch’a cha*
*Víbora mano de metate*		*cu*	*ya na*
*Víbora escorpión*	*Abronia* sp.	*cu*	*ya da*
Coral snake	*Micrurus* sp.	*cu*	*yab yuu*
*Víbora sorda*	*Crotalus* sp.	*cu*	*yab doo*
Boa	*Boa constrictor*	*cu*	*y’u du*
*Víbora ratonera*	*Crotalus* sp.	*cu*	*que cuoó*
*Víbora lechera*	*Drymarchon melanurus*	*cu*	*ji ya*
Bright snake	*Epictia* sp.	*cu*	*na ma*
Víbora bejuquera	Colubridae	*cu*	*ndu cu*

We could not record terms for specifically grouping amphibians (frogs, toads, and salamanders). Reptiles are grouped into three categories *yuca tasa* (crocodiles), *y’ati* (lizards), and *cu* (snakes) (Table 2).

Birds in general are grouped with the term *y’ada* (Table 3). However, for naming specifically some of them, the term is not always used. Some birds are grouped with the term cu’; others like roadrunner and woodpeckers are grouped with the prefix *rita* or *dita*. Several avian groups used the prefix *i’*; the prefix *i’yun* groups pheasants and turkeys; *i’m*, owls, macaws (*Ara militaris*), chacalaca (*Orthalis vetula*), and the hawk *Accipiter bicolor*. The prefix *s’i* groups scavenger birds like buzzards and crows (Table 3). Hummingbirds are called *tindu* and domestic hens, and chicken are named with the prefix *tu*.

**Table 3:** Examples of Cuicatec nomenclature and classification of birds, based on fieldwork in San Lorenzo Pápalo and reports from [49] in Santa María Pápalo, Oaxaca

Common name	Scientific name	Classificatory term	Specific term
Birds	All birds	*y’ada*	
Blue bird		*y’ada*	*cue*
Swallow	*Hirundo rustica*	*y’ada*	*y’inda*
Quail	*Colinus virginianus*	*y’ada*	*yeco*
*Sadía*	*Cyanocorax* sp.	*y’ada*	*chi cuili*
Calandra lark	*Icterus* sp.	*y’ada*	*til cuó*
Trogon	*Trogon* sp.	*y’ada*	*chava’a*
Group *Cu’*			
Heron	*Bubulcus ibis*	*cu’*	*anda*
Dove	*Zenaida asiatica* *Zenaida macroura*	*cu’*	*chun*
*Zanate*	*Quiscalus mexicanus*	*cu’*	*‘iya*
*Urraca*	*Corvus corax*	*cu’*	*cubé*
Group *Rita*			
Roadrunner	*Geococcyx velox*	*rita*	*d’a ndi*
Woodpecker	*Melanerpes hypopoleus*	*rita*	*´quian*
Woodpecker	*Melanerpes fromicivorous*	*rita*	*tin guoo*
Group *i’*			
Turkey	*Meleagris gallopavo*	*i’ yun*	*ya*
*Chachalaca*	*Ortalis vetula*	*i’nga*	*cha*
Hawk	*Accipiter* spp.	*i’ ya*	
Macaw	*Ara militaris*	*i’va*	
Owls	*Tyto alba*	*i’mi*	
Group *s’i*			
*Zopilote*	*Coragyps atratus*	*s’i*	*cu*
*Zopilote cabeza dorada*	*Cathartes aura*	*s’i*	*cu cuá*
*Quebrantahuesos*	*Polyborus plancus*	*s’i*	*cu nanda*

Among mammals, the Cuicatec classify four main groups named *iyu*, *y’e*, or *y’aa* (the large carnivores like cougar and coyote) and *y’u* (Table 4). Other groups receive specific names, as it is the case of bats (*be’e*), moles (*yi ngu’iné*), mice and rats (*ti ’u di*), and skunks (*s’ima*)—*Spilogale putorius* is named *s’ima ajo*, while *Mephitis macroura* is named *s’ima ata*. Deer are called *it cheno* (*Odocoileus virginianus*) and pecari *cu cheno****.***

**Table 4:** Examples of Cuicatec nomenclature and classification of mammals, based on fieldwork in San Lorenzo Pápalo and reports from [49] in Santa María Pápalo, Oaxaca

Common name	Scientific name	Classificatory term	Specific term
Group *y’aa*			
Cougar	*Felis concolor*	*y’aa*	*iuùo’o*
Ocelotl	*Leopardus pardalis*	*y’aa*	*va*
Coyote	*Canis latrans*	*y’aa*	*ga ta*
Group ‘*i-yu*			
Squirrel	*Sciurus* spp.	*‘i-yu*	
Mole	*Thomomys umbrinus*	*‘i-yu*	
Weasel	*Mustela crenata*	*‘i-yu*	
Group *y’u*			
*Temazate*	*Mazama temama*	*y’u*	*du*
Fox	*Urocyon cinereoargenteus*	*y’u*	*ne*
Cacomistle	*Bassariscus astutus*	*y’u*	*ne sangá*
Badger	*Nasua narica*	*y’u*	*b’ama*
Badger	*Nasua narica*	*y’u*	*b’yoo*
Racoon	*Procyon lotor*	*y’u*	*bi*
Anteater	*Tamandua mexicana*	*y’u*	*bi ndii*
Zerete	*Dasyprocta mexicana*	*y’u*	*cue*

## Use of arthropods

### Insects

#### Collecting of the *chicatana* (*Atta mexicana*)

**Fig. 1:**
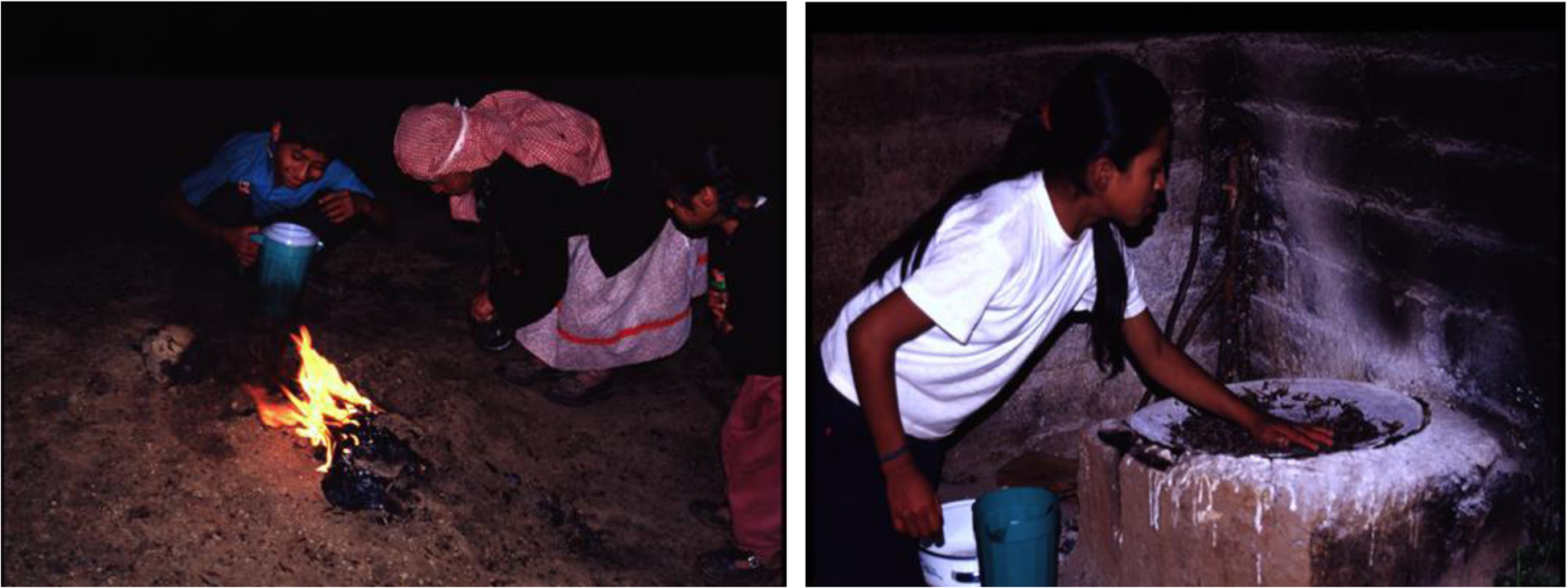
The *chicatana* ant (*Atta mexicana*), one of the most valuable edible resources from the wild. **a** Aspect of the *chicatana* dance during the nights by the end of June with the participation of the family with fires near the anthills. **b** Roasting of *chicatana* ants in a *comal*, which is the round hotplate made of clay, that is heated directly on fire

*Chicatanas* (*Atta mexicana*) are highly appreciated edible ants named *san’go* (Fig. 1). Our survey recorded 83.3% of people consuming them. Ants of this species are present the year round, but it is only during the feasts of San Juan and San Pedro (24 to 29 of June), with the starting of the rainy season, when it happens a massive flight of winged ants (out of the anthills) for dispersal. When these dates approach, people of San Lorenzo start to observe the enlargement of anthills’ mouths and they prepare the “*chicatana* dance” (Fig. 1a), which consists in nocturnal visits to the anthills to collect the winged ants. People distinguish three types of ants, the *hormiga arriera* (worker ants) or *t’u cudi*, which are small winged ants that are identified as signs that the *chicatanas* are coming, and the *chicatanas san’go* which are winged larger ants.

If the *chicatanas* start their flight, people collect them in the anthill’s mouths, with their hands, and put the ants in pots (on average, 1 L per household). Ants may be stored while maintained alive supplying to them avocado leaves as food.

The most popular form of preparing these ants is the *chicatana* sauce, by toasting and then grinding the ants (Fig. 1b). For each cup of ants, people add one tomato, one chili pepper, one garlic clove, and salt. This food is protein rich [28], and people consider that eating it cures the *yu du d’u*, the way they call sadness or depression. These ants are collected mainly in the warmer habitats (tropical dry forest). Consumption of *chicatanas* contributes to local people valuable nutriments in diet.

#### The *jonote* worm or *cuetla* (*Arsenura armida*)

**Fig. 2:**
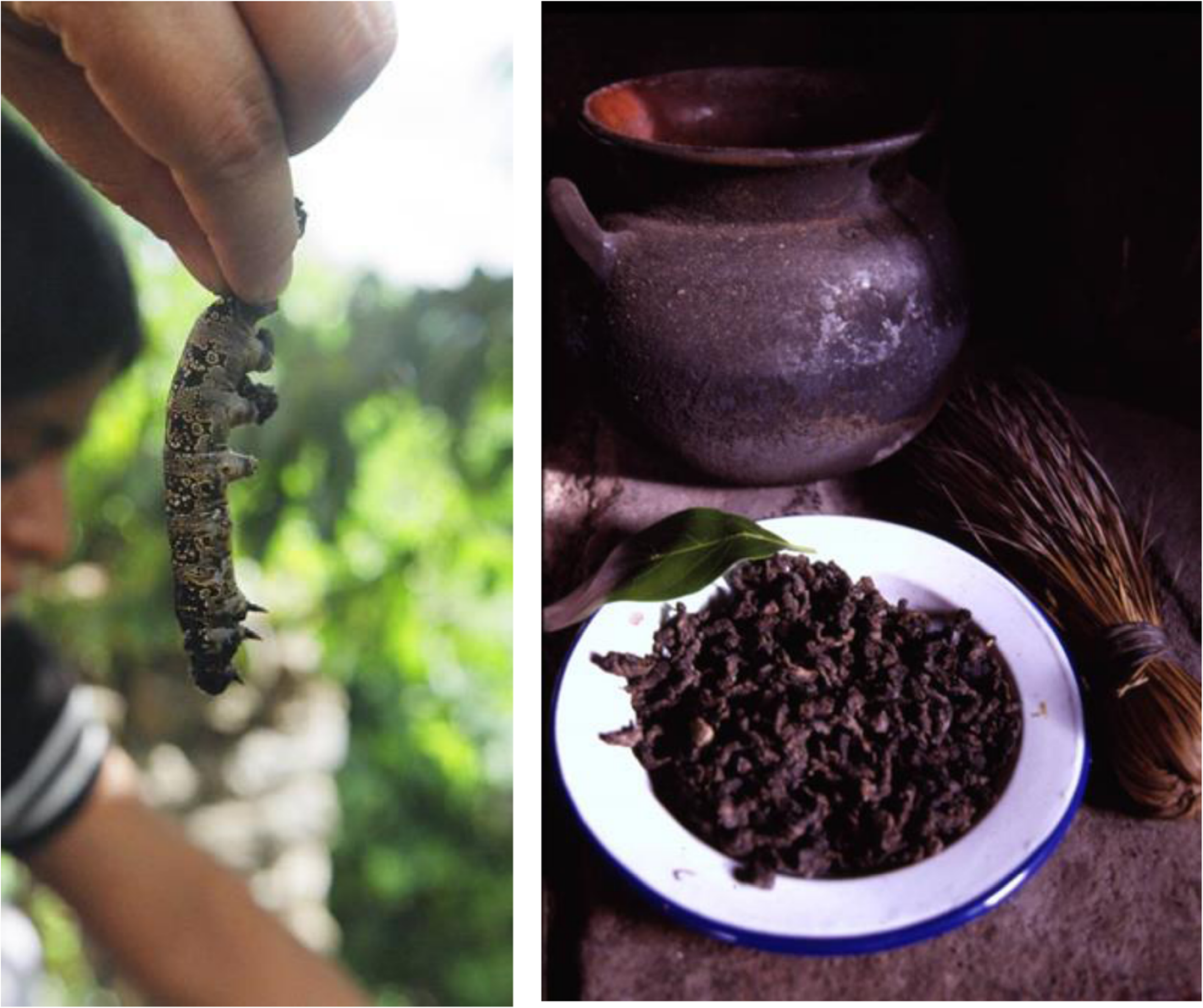
The *cuetla Arsenura armida*. Aspect of collecting of larvae and it is the food prepared after cleaning, boiling, and frying the animals

The *jonote* worm is named *doo ya’a dacua* in Cuicatec (the term derives from *do ‘o* = worm, and *y’aa dacua* = *jonote* tree, *Heliocarpus velutina*). The worms are the larvae of the butterfly *Arsenura armida* (Fig. 2a), which are available from July to September, living on the branches and leaves of *H. velutina*. These worms are gathered by 83.30% of people surveyed, just picking the worms from trees, although 10% of people said to cut the trees to make the collecting easier (Fig. 2a). Nearly 90% of gatherers said that they use to collect only the large worms, leaving on the trees those of small and intermediate sizes. For preparing these worms, people cut their heads to remove their inner organs, then they wash their bodies with water, lemon, and salt (some people use to boil them with water, lemon, and avocado leaves). Later, the prepared worms are dried to sun for several days and finally roasted (Fig. 2b).

Sampling of vegetation allowed identifying on average 17 *jonote* trees in 500 m^2^ transects. On average, 13 had worms (76.5%) with 28 ± 7.8 worms per tree. We identified nearly 500 *jonote* trees, that is, approximately 14,088 worms, per hectare. Every household consumes 373 ± 372.8 worms per year, which means that one hectare of tropical dry forest may provide approximately the amount consumed by 37 households; therefore, all people of San Lorenzo Pápalo satisfy their requirement of *cuetlas* with 3.95 hectares of tropical dry forest. These edible larvae have been reported to provide valuable nutriments to diet [28].

#### Collecting of honey

Based on the survey conducted, 76% of people of San Lorenzo Pápalo collect honey throughout the year. This activity is not planned but rather opportunistic, when other activities are carried out. Nearly 41.60% of people surveyed said to collect honey in both temperate and warm areas, whereas 58.30% said they collect honey in the warm area. Table 5 shows data on the frequency of honey collecting. When bee nests are within trees, they cut the tree. In addition to the European bees, the Cuicatec people identify the bumble bees, called *t’u iñó*.

**Table 5:** Percentage of people surveyed that reported practicing gathering of honey in the wild in San Lorenzo Pápalo (*N* = 30 households interviewed)

Frequency of honey gathering	Percentage of people
Every 6 months	9.00
Once per year	50.00
Once every 2 years	4.50
Once per three or more years	9.00
Do not practice this activity	27.50

### Reptiles

**Fig. 3:**
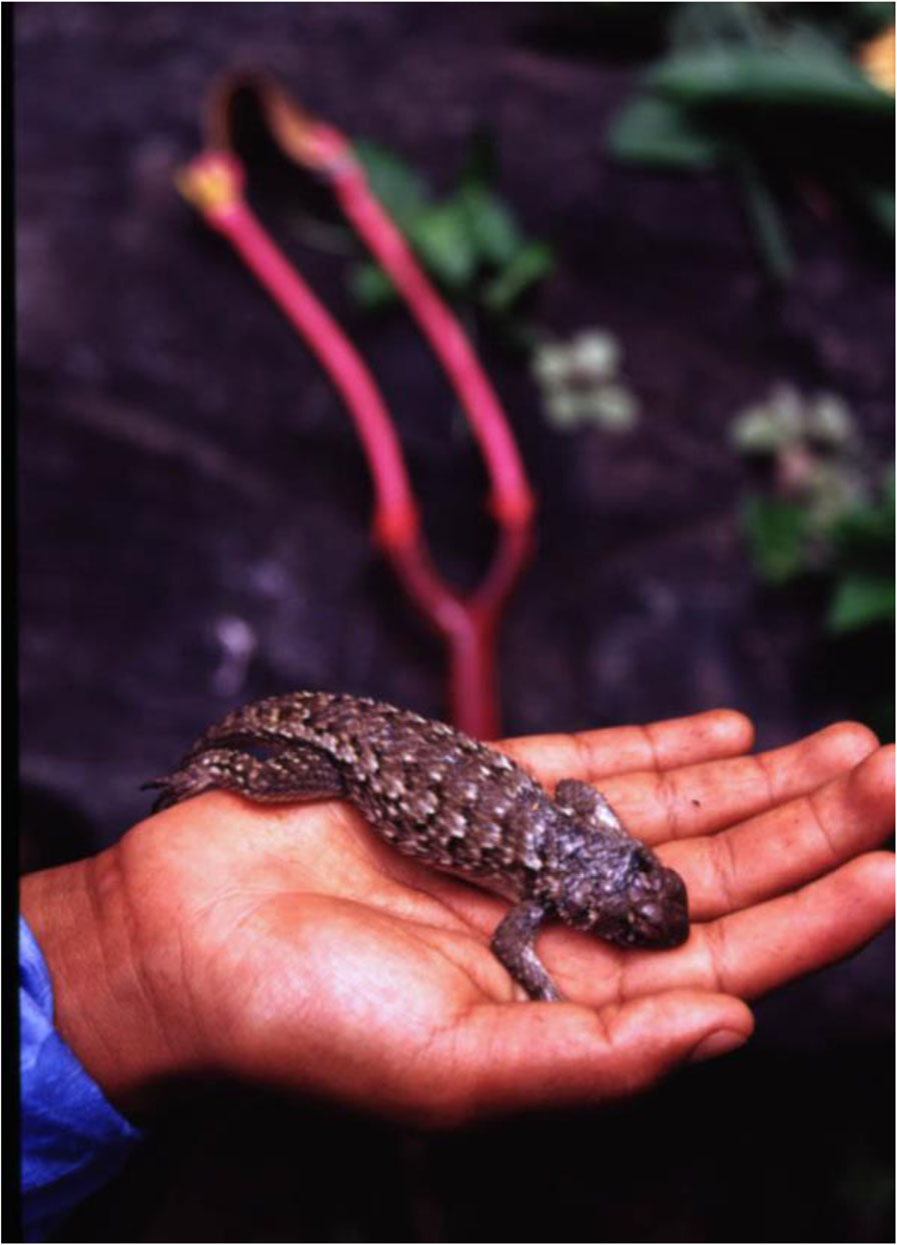
The lizard *Sceloporus grammicus*, the main edible reptile consumed by the Cuicatec people, prepared in soups with flowers of the columnar cacti *Pilosocereus chrysacanthus* and *Stenocereus pruinosus*

People of San Lorenzo Pápalo consume the lizard *Sceloporus grammicus* called *y’ati cua*, which is hunted in the tropical dry forest (Fig. 3). Nearly 76% of people said to consume this species during April and May (on average, 30 ± 10 lizards per year per household) preparing a soup mixing the lizard with flowers of *Pilosocereus chrysacanthus* and *Stenocereus pruinosus*, or it is consumed after being roasted.

Nearly 53% of people surveyed said that they occasionally consume iguanas, recognizing the green iguana (*Iguana iguana)* and the black one (*Ctenosaura pectinata*), preparing these animals in “mole” sauces, cooked in underground ovens or roasted.

Rattle snakes (several species) were recognized to have medicinal properties by 73% of people surveyed; however, people’s perception is that these animals are scarce within the territory of San Lorenzo Pápalo. Meat and tallow grease are used as medicine against epilepsy, gastric ulcer, and kidney illnesses, whereas the grease is used as ointment to alleviate pain caused by rheumatism. The meat is prepared roasted, whereas the grease is mixed with camphor resin.

### Birds

Among birds occurring in the area, 56.60% of local people mentioned to chase the mountain doves (*Zenaida macroura* and *Z. asiatica*), 46.60% of people said to chase the torcaza dove (*Leptorilla cassinrii*), 26.60% chase chachalacas (*Ortalis vetula*), 26.60% the quail (*Colinus virginianus*), 16.60% the macaws (*Ara militaris*), 6.60% the small doves (*Columbina* sp.), and 3.30% the hummingbirds (several species, among them *Amazilia violiceps* and *A. latirostris*).

*Zenaida macroura* and *Z. asiatica* were recorded to be nurtured as pets in several homes of the village. People mentioned that when these animals are restless announce the arrival of visits. In one home, we identified the presence of three individuals of *Melanerpes formicivorus*. Nurturing all the species mentioned involves a deep knowledge about their habits to take care of them from chicks to adults.

Feathers of hummingbirds are used to cure epilepsy. People said that the macaw beaks (*Ara militaris*) were used in the past for inducing childbirths, but this use has been abandoned. Local people have the belief that crossing the way by a roadrunner (*Geococcyx velox*) is a sign of bad luck and that resting of an owl on a house’s roof indicates that somebody is going to die. Other birds like the *sadías* (*Cyancorax* sp.) announce problems.

### Mammals

Species of mammals in Table 6 are the most used and preferred, while those referred to in Table 7 are those more commonly consumed and in higher amounts. The surveys carried out indicate that 56% of people do not practice hunting, whereas the remaining 44% carry out this practice using rifles and shotguns. People use to go hunting once every 1 or 2 months, and however the irregular periodicity of this practice, it represents an important source of animal proteins for local people. Consumption of domestic animals (chickens, turkeys, goats, and pigs) is occasional, mainly during feasts.

**Table 6:** Percentage of mention of mammals hunted in San Lorenzo Pápalo, Oaxaca (*N* = 30 households interviewed)

Species	Percentage of mention
Badger (*Nasua narica*)	62.50
Deer (*Odoicoleus virginianus*)	56.25
Squirrel (*Sciurus* spp.)	56.25
Fox (*Urocyon cinereoargenteus*)	43.25
Rabbit (*Sylvilagus* sp.)	43.25
Armadillo (*Dasypus novemicinctus*)	37.50
Skunks (*Spilogale putorus, Maphitis macrura*)	25.00
Opossum (*Didelphys virginiana*)	18.75
Racoon (*Procyon lotor*)	12.50
Leoncillo o tigrillo *(Leopardus pardales)*	12.50
Zerete (*Dasyprocta mexicana*)	12.50
Jabalí (*Tayassu tajacu*)	6.25

**Table 7:** Number of animals of mammal species documented to be consumed on average ± standard deviation per household per year in San Lorenzo Pápalo (*N* = 30 households interviewed)

Animals hunted	Animals consumed per household	Animals consumed by the whole community
Squirrel (*Sciurus* spp.)	1.10 ± 0.34	83.75
Badger (*Nasua narica*)	0.48 ± 2.70	36.25
Deer (*Odocoileus virginianus*)	0.13 ± 1.21	10
Fox (*Urocyon cinereoargenteus*)	0.10 ± 0.40	7.50
Skunks (*Spilogale putorus, Maphitis macrura*)	0.06 ± 0.36	5
Rabbits (*Sylvilagu*s sp.)	0.03 ± 0.18	2.50
Armadillo (*Dasypus novemicinctus*)	0.03 ± 0.18	2.50

Nearly 53.30% of people interviewed indicated medicinal uses of one or more mammal species. Grease and skin of coyote are medicines; grease is used to alleviate back and kidney pains, whereas the skin is used to calm anxiety or even a fit of madness.

Skunks were the most mentioned medicinal animals in San Lorenzo; their meat and grease are used against epilepsy, kidney illnesses, and cough. Glandules causing the strong odor of these animals are used to protect the maize fields against badgers.

Grease, tail, and antlers of deer are used as medicine for treating an illness called *motolín*, which appears to be an allergic reaction. Tails of opossums and armadillos are used for inducing births; for that purpose, tails are cooked in a kind of soup.

#### The regulation of hunting

Most people of San Lorenzo recognize that the Federal Government forbids hunting after the decree of the Biosphere Reserve Tehuacán-Cuicatlán; however, the communitarian regulations allow it. Although no formal written rules are available, hunters recognize the periods of reproduction of several species and they avoid hunting of females pregnant or with cobs. Animals like squirrels and rabbits are hunted throughout the year.

**Fig. 4:**
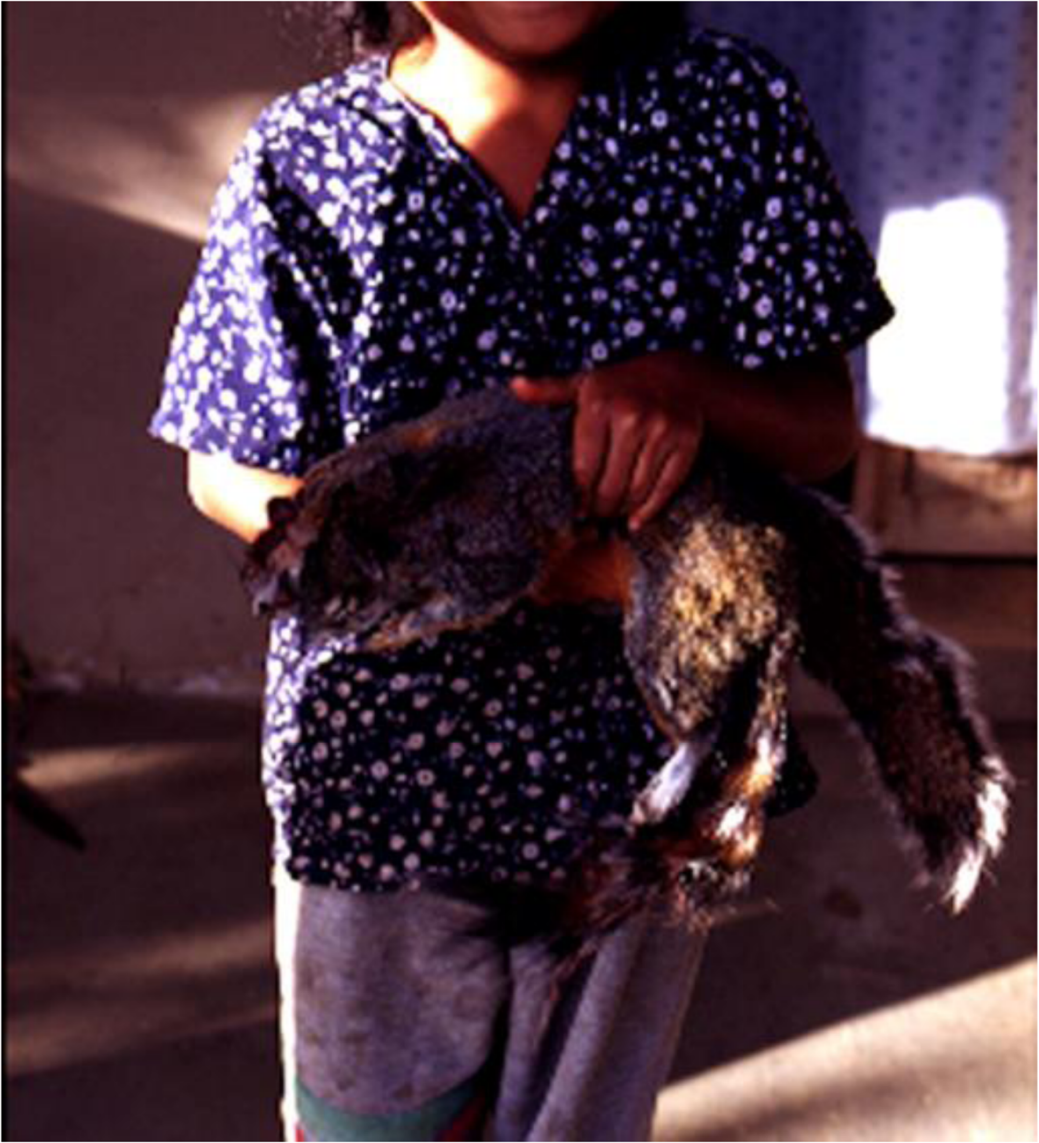
The squirrels *Sciurus* spp. Among the most frequently consumed animals by the Cuicatec people of San Lorenzo Pápalo

**Fig. 5:**
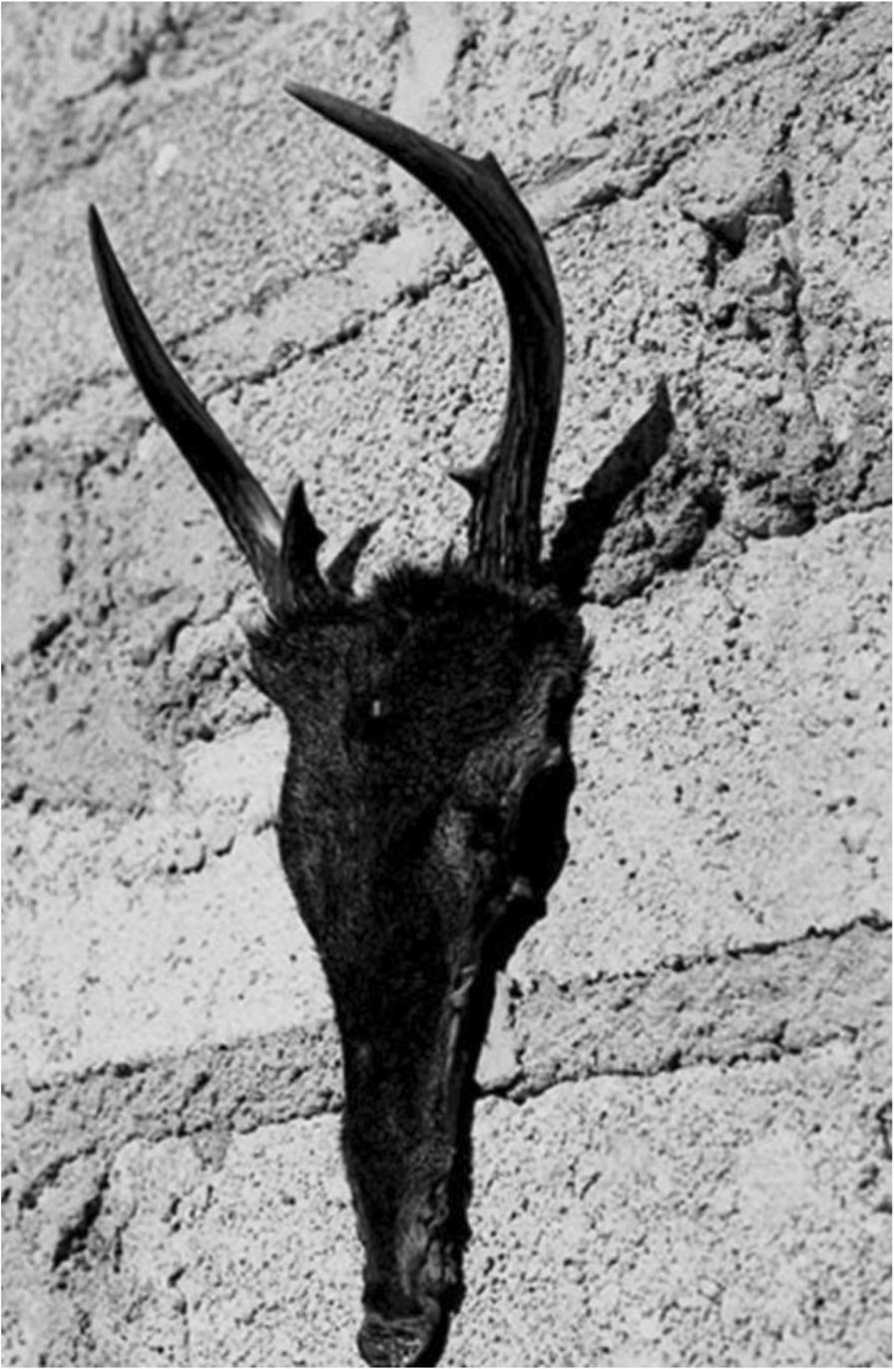
The deer *Odocoileus virginianus*, the most preferred animal as food but also the most difficult to be hunted in San Lorenzo Pápalo

Nearly 52.63% of people surveyed coincided that the most appreciated meat is that from deer, 21% of people interviewed said that meat of all animals from the mountain is good, and the rest said to prefer different species. However, according to the surveys, people more frequently consume meat of squirrels (46.66%) and badger *Nasua narica* (46. 66%). According to people, the easiest species to hunt are squirrels (23% of people surveyed; Fig. 4) and badger (17.64% of people surveyed); however, 29.41% of people said that all animals are difficult to be hunted, but the most difficult are deer (81.25% of people; Fig. 5).

## Discussion

### Cuicatec nomenclature of fauna

Our data from nomenclature provide interesting criteria for analyzing the Cuicatec classification of animals and support some ideas from linguistic studies. For instance, studies of several languages related to the Mixtec, among them the Cuicatec [46], identified several names of insects, avian, and mammals starting with the term *i* or *‘i* followed by a consonant, which suggests a historic relation with the term *iti* recorded in our study. A second group starts with *y’a*, *y’e*, and *y’u*, suggesting that the prefixes *‘i* and *y’* are reminiscences of a prefix distinguishing certain animals in the proto-Cuicatec or the proto-Mixtecan languages [46]. Our nomenclatural study supports this thinking. De Ávila [46] also found that other ethnozoological terms in Cuicatec start with consonant, like in the cases of *cu* (relative to the Mixtec term *coo* naming snakes), and the terms *nd’*, *san*, *sti*, *s’i*, which classify several groups of animals as found in our study. Similar with facts registered by De Ávila [46], we found the category *y’aa*, which includes wild beasts or large size carnivorous, which according to this author groups animals not according to their morphological similarities but because of their symbolic affinities.

### Role of animals in Cuicatec subsistence

#### Insects

According to Ramos-Elorduy and Pino [52], nearly 2000 species of edible insects have been reported in the world. For Mexico, more than 500 species of edible insects have been reported, mainly Coleoptera, Hymenoptera, Hemiptera, and Orthoptera, 195 of which are commercialized in markets [53, 54]. These animals are important sources of nutrients (high levels of proteins, aminoacids, vitamins, and minerals) in diet of indigenous peoples of Mexico since ancient times [55–57].

In San Lorenzo Pápalo, consumption of insects is mainly directed to the *jonote* worm and the *chicatana* ants, in considerable amounts. Our study provides a first approach to evaluate the sustainability of using this resource. We identified that according to the average density of *Heliocarpus velutina* in the tropical dry forests of the area, approximately 4 ha of this vegetation type would be sufficient to satisfy the annual need of the whole village on this resource. Such area is a small portion of the cover of this vegetation type within the territory of the community. However, the temporal availability and amounts of *jonote* worms may vary among years; therefore, a long-term monitoring of the availability of these resources would be necessary, as well as more detailed studies about population dynamics of this species. Such information would be helpful to evaluate the effect of larvae gathering by people and interannual effects of climate variation and predation of worms by other animals.

#### Reptiles

Lizards and iguanas are the most important reptiles consumed by people of San Lorenzo Pápalo. It appears to be particularly high the number of lizards hunted every year, and although we did not study the distribution and abundance nor population ecology of this species, it is possible to warn about such rate. Ecological studies would be therefore a priority for providing criteria about management planning. Consumption of iguanas appear to be markedly lower than that of lizards, but more precise studies about their hunting and availability would be recommendable for planning their sustainable use.

#### Birds

Consumption of birds is an on-going practice; it appears to be very low since during our study we did not record any household consuming wild birds. However, people referred to seven edible species. Although no ecological data are still available, it appears that impact of hunting birds would be low. Endangered species like the macaw *Ara militaris*, which is protected through the Appendix of CITES, the official Mexican regulations (NOM-059-SEMARNAT-2010) and the BirdLife International (BLI 2000) [58], does not appear to be threatened because of its medicinal use since childbirths are currently attended by physicians in the health center; alternatively, other traditional remedies such as using of tails of opossums and armadillos are more common.

#### Mammals

Among the mammals hunted in San Lorenzo Pápalo, those within a conservation category are the zerete (*Dasyprocta mexicana*), which is in the list of IUCN as a species of low risk, and the badger (*Nasua narica*) in the category III of CITES. The most hunted mammals are squirrels, badgers, and deer; but there are no ecological studies for the region documenting their abundance, and it is therefore difficult to say anything about the magnitude of risk of their hunting. However, it is important to recognize that populations of these mammals are possibly affected by hunting and other activities disturbing their habitat. Therefore, ecological studies would be important for providing more specific criteria for constructing regulation systems at local and regional levels in order to achieve goals of sustainable use of these resources.

The traditional Cuicatec ecological knowledge in San Lorenzo Pápalo is particularly outstanding in relation to aspects on distribution, abundance, natural history, habits, habitats, behavior, reproduction patterns, among other issues. Although there are no formal written regulations, most people surveyed respect some criteria for regulating hunting in relation to reproduction, areas of distribution, and abundance.

Consumption of meat from wild animals continues being an important source of proteins for local people. Domestic animals are raised for occasional consumption in feasts and for interchange. But this pattern is changing since the commercialization of chicken meat is locally increasing. The main trend identified is the substitution of wild meat by chicken meat.

However, consumption of wild meat continues being part of the Cuicatec cultural identity; hunting is part of this identity and a way of transmitting knowledge about local environments and fauna to the new generations. In other words, it is an important part of constructing and transmitting traditional ecological knowledge.

Ethnozoology has been poorly developed in the Tehuacán Valley, and it is, however, highly important for the regional management programs of wildlife conservation. Particularly important will be, therefore, social-ecological studies directed to provide criteria for sustainable management, conservation, and recovering of regional flora and fauna.

### Management of fauna by the Cuicatec

Information about local and regional management strategies of fauna is still scarce, and the inventory of local experiences is a priority for conservation purposes. Except for turkeys, all domestic animals of the village are species introduced from the Old World. Regional wild animals are exceptionally nurtured, mainly maintained as pets, without breeding throughout generations. However, the local experiences of nurturing wild animals are important in conservation programs. Also important are the strategies reflected in the communitarian regulations described. However, details about this issue are yet to be investigated more deeply in a larger sample of cultural and ecological contexts.

Interactions between people and plants in the Tehuacán Valley include nearly 2000 species, from simple gathering to domestication [1, 2]. Management of plants beyond simple gathering involves nearly 800 species [1, 2]. These forms of interactions are less clear and scarcer for animal species. But these are happening. Interactions with most animal species can be characterized as simple extraction (simple gathering of insects or hunting). However, other forms of extraction involve careful strategies involving knowledge of behavior, distinction between male and female organisms, and designing strategies and organizing specific activities for collecting or hunting them. These are for instance the cases documented for the *chicatana* ants and deer hunting. Agreements among households for respecting areas where they carry out their activities and communitarian regulations for protecting the activities and animals are both expressions of interactions more than “simple”. Special care of trees where the *cuetla* larvae live and indications to collectors, which may or may not have the right of accessing to them, are all expressions of especial interactions with a valuable resource. Husbandry of some birds, rabbits, and other mammals is another form of interactions. Finally, care, time invested, and practices of artificial selection are all active among the several domesticated species. The review conducted by Zarazúa [14] about types of interactions between Mesoamerican peoples and fauna is an important reference. It allows analyzing general patterns of relations between humans and animals that are generally similar to those identified for plants. These patterns should be more studied but make general sense to hypotheses proposed by Zeder [34, 35]. Our research team explores such a gradient of intensity of interactions between people and plants, animals, mushroom species, and even with communities of microorganisms managed through management techniques of fermented products. But all these interactions between peoples and the different groups of organisms require more deeply documentation, based on case studies that eventually will make possible characterizing general patterns. This study is a first step in such a direction in an important region for understanding the origins of domestication and agriculture: The Tehuacán Valley.

## Conclusions

The Cuicatec TEK of animals is reflected in the extensive nomenclature and classification of animal groups, some of them coinciding with the formal zoological taxonomy, some others based on cultural aspects and local views and beliefs. Wild animals continue being important elements for the Cuicatec subsistence, complementing diet based on agricultural products and domestic animals. Their use as medicine is still reported, but according to people, these have been substituted by medical treatments in the local health center. Particularly important is the general view of local people and communitarian authorities about the need to conserve forests to ensure the occurrence of animals, which are valued as part of nature, the beauty of their territory and their culture. Local regulations are the main and most effective actions for conserving fauna in the study area, and the local practices of management are valuable experiences potentially helpful in conservation programs. Intensity of interactions is not linear; it is influenced by cultural and economic values of animals, as well as their scarcity and perception of risk to disappear.

## Data Availability

Data that support the analysis and additional data are provided in Tables 1, 2, 3, 4, 5, 6, 7, and 8.

## Appendix

**Table 8:** Animal resources found as archaeological remains in the Tehuacan Valley (based on [17]), and current uses recorded in this study. Phases: Ajuereado^1^, El Riego^2^, Coxcatlán^3,^ Abejas^4^, Purrón^5^, Ajalpan^6^, Santa María^7^, Palo Blanco^8^, Venta Salada^9^. Uses: 1 = edible, 2 = medicinal

Species	Common name	11,000^1^	8500^2^	6200^3^	4900^4^	3200^5^	2550^6^	2250^7^	1100^8^	880^9^	Current use
Insects											
*Atta mexicana*	chicatana										1
*Arsenura armida*	jonote worm										1
Reptiles											
*Kinosternon integrum*	turtle		X	X	X		X		X		
*Gopherus berlandieri*	Pleistocene turtle	X									
*Iguana iguana*	Green iguana							X	X	X	1
*Ctenosaura pectinata*	Black iguana							X	X	X	1
*Ameiva Udulata*	lizzard	X	X	X			X	X	X	X	
*Sceloporus grammicus*	lizzard										1
*Crotalus basiliscos*	Rattle snake								X		1,2
Birds											
*Anas cyanoptera*	Cinnamon duck										
*Colinus virginianus*	Codorniz	X	X					X			1
*Meleagris gallipavo*	turkey										
*Charadrius vociferus*	Chichicuilote										
*Zenaida asiática*	Dove		X		X		X		X		1,3
Zenaida macroura	Dove										1,3
*Leptorilla cassinrii*	Torcaza										1,3
*Columbina passerina*	Dove		X		X		X		X		
*Tyto alba*	Owl			X							
*Caprimulgus ridgwayi*	Chotacabras									X	
*Chordeiles acutipennis*	Chotacabras										
*Corvus corax*	Crown							X		X	
*Ara militaris*	Macaw										2
Diferentes especies	Hummingbird										2
Mammals											
*Didelphys marsupialis*	Opposum										2
*Artibeus jamaicensis*	bat									X	
*Eptesicus fuscus*	bat									X	
*Lepus callaotis*	Hare										
*Sylvilagus* spp.	Rabbit	X									1
*Spermophilu*s sp.	Squirrel	X									
*Sciurus* spp.	Squirrel										1
*Hetorogeomys* sp.	Mouse										
*Cratogeomys* sp.	Mouse										
*Dipodomys* spp.	Mouse										
*Lyomis* spp.	Mouse	X	X		X		X	X	X	X	
*Peromiscus* spp.	Mouse	X	X	X	X		X	X	X	X	
*Neotoma* sp.	Packrat	X	X	X	X		X	X	X	X	
*Canis* sp.	Coyote	X	X	X			X	X		X	2
*Urcyon* spp.	Fox	X	X	X	X				X	X	
*Bassariscus astutus*	Cacomixtle	X									
*Nasua narica*	Badger										1
*Procyon lotor*	Raccon				X			X			1
*Spilogale* sp.	Skunk	X	X	X	X	X		X	X	X	1,2
*Conepatus* sp.	Skunk	X	X	X	X	X		X	X	X	
*Maphitis macroura*	Skiunk										1,2
*Felis concolor*	Cougar		X					X			
*Lynx rufus*	gato montes		X								
*Pecari tajacu*	jabalíes		X	X	X		X	X	X	X	1
*Aguti paca*	tepezcuincle										1
*Odoicoleus virginianus*	Deer	X	X	X	X		X	X	X	X	1,2

## Abbreviations

UNAM: Universidad Nacional Autónoma de México
SEMARNAT: Secretaría de Medio Ambiente y Recursos Naturales
CONANP: Comisión Nacional de Áreas Naturales Protegidas
CONACYT: Consejo Nacional de Ciencia y Tecnología
DGAPA: Dirección General de Asuntos del Personal Académico
